# Transcriptome resources and functional characterization of monoterpene synthases for two host species of the mountain pine beetle, lodgepole pine (*Pinus contorta*) and jack pine (*Pinus banksiana*)

**DOI:** 10.1186/1471-2229-13-80

**Published:** 2013-05-16

**Authors:** Dawn E Hall, Macaire M S Yuen, Sharon Jancsik, Alfonso Lara Quesada, Harpreet K Dullat, Maria Li, Hannah Henderson, Adriana Arango-Velez, Nancy Y Liao, Roderick T Docking, Simon K Chan, Janice EK Cooke, Colette Breuil, Steven JM Jones, Christopher I Keeling, Jörg Bohlmann

**Affiliations:** 1 Michael Smith Laboratories, University of British Columbia, 2185 East Mall, Vancouver, British Columbia V6T 1Z4, Canada; 2 Department of Biological Sciences, University of Alberta, Edmonton, Alberta T6G 2E9, Canada; 3 British Columbia Cancer Agency Genome Sciences Centre, Vancouver, British Columbia V5Z 4E6, Canada; 4 Department of Wood Sciences, University of British Columbia, 2424 Main Mall, Vancouver, British Columbia V6T 1Z4, Canada

**Keywords:** Conifer defence, Pine oleoresin, Terpenoid biosynthesis, Metabolite profile, Prenyl transferase, Cytochrome P450, Conifer genome

## Abstract

**Background:**

The mountain pine beetle (MPB, *Dendroctonus ponderosae*) epidemic has affected lodgepole pine (*Pinus contorta*) across an area of more than 18 million hectares of pine forests in western Canada, and is a threat to the boreal jack pine (*Pinus banksiana*) forest. Defence of pines against MPB and associated fungal pathogens, as well as other pests, involves oleoresin monoterpenes, which are biosynthesized by families of terpene synthases (TPSs). Volatile monoterpenes also serve as host recognition cues for MPB and as precursors for MPB pheromones. The genes responsible for terpene biosynthesis in jack pine and lodgepole pine were previously unknown.

**Results:**

We report the generation and quality assessment of assembled transcriptome resources for lodgepole pine and jack pine using Sanger, Roche 454, and Illumina sequencing technologies. Assemblies revealed transcripts for approximately 20,000 - 30,000 genes from each species and assembly analyses led to the identification of candidate full-length prenyl transferase, TPS, and P450 genes of oleoresin biosynthesis. We cloned and functionally characterized, via expression of recombinant proteins in *E. coli*, nine different jack pine and eight different lodgepole pine mono-TPSs. The newly identified lodgepole pine and jack pine mono-TPSs include (+)-α-pinene synthases, (-)-α-pinene synthases, (-)-β-pinene synthases, (+)-3-carene synthases, and (-)-β-phellandrene synthases from each of the two species.

**Conclusion:**

In the absence of genome sequences, transcriptome assemblies are important for defence gene discovery in lodgepole pine and jack pine, as demonstrated here for the terpenoid pathway genes. The product profiles of the functionally annotated mono-TPSs described here can account for the major monoterpene metabolites identified in lodgepole pine and jack pine.

## Background

The mountain pine beetle (MPB; *Dendroctonus ponderosae* Hopkins) epidemic has affected an area of more than 18 million hectares of lodgepole pine (*Pinus contorta* Douglas) in western Canada [1,2]. As the geographic range of the epidemic has expanded eastward beyond the Rocky Mountains, MPB has become a threat to jack pine (*Pinus banksiana* Lamb.), the dominant pine species of Canada’s boreal forest. MPB has successfully colonized jack pine in the lodgepole pine/jack pine hybrid zone [3]. The beetle may have similar reproductive success and survival on lodgepole pine and jack pine [4,5]. While the suitability of jack pine as a major host for MPB and the ability of these trees to survive during outbreak of MPB are unknown, host range expansion of MPB from lodgepole pine into jack pine forests has heightened the risk of increased environmental impact of the MPB epidemic.

Successful MPB colonization of pine trees occurs when host defences are weakened or when mass attack by MPB and associated fungi overwhelm host defences [6,7]. One of several defence mechanisms employed by conifer trees is the production of terpenoid oleoresin, which acts as a physical and chemical barrier against insect and pathogen attack [8-10]. Oleoresin is stored in resin ducts of conifer bark, wood and needles and is primarily composed of C_10_ monoterpenes and C_20_ diterpene resin acids [11-13]. Monoterpenes are involved in the constitutive and induced defence response of lodgepole pine against adult bark beetles, brood, and beetle-associated fungi; however, the effectiveness of monoterpenes against MPB is variable and may depend on beetle population density [7,14]. During dispersal flight, adult MPB also exploit pine monoterpenes as cues to identify suitable host trees and as precursors for their pheromone biosynthesis [15]. Insect-associated fungi also play a role in the MPB epidemic. The MPB-associated pine pathogen *Grosmannia clavigera* has molecular mechanisms for transport and tolerance of certain monoterpenes [16]. In culture, *G. clavigera* can use the monoterpene (+)-limonene as a carbon source [17].

Lodgepole pine trees respond with induced accumulation of monoterpenes after MPB attack [18] or after inoculation with *G. clavigera* (also previously referred to as *Ceratocystis clavigera* or *Europhium clavigerum*) [7,19,20]. Lodgepole pine survival is positively correlated with high levels of oleoresin secretion after fungal inoculation [21], and lodgepole pine trees with high levels of monoterpenes had decreased frequency of attack at low MPB population density [7]. Previous analyses identified (−)-β-phellandrene and (−)-β-pinene as the most abundant oleoresin monoterpenes of lodgepole pine, with moderate levels of (+)-3-carene and α-pinene also detected [7,22-25]. The primary monoterpenes of jack pine oleoresin were reported as α-pinene and 3-carene, with lower amounts of β-pinene, limonene and myrcene [26,27]. Comparison of the volatile emissions showed that lodgepole pine emitted higher amounts of β-phellandrene and β-pinene, while jack pine emitted higher levels of α-pinene and 3-carene [27]. Crude extracts from xylem of lodgepole pine saplings contain terpene synthase (TPS) activity for biosynthesis of (−)-β-phellandrene, (+)-3-carene, (−)-β-pinene and (−)-sabinene, consistent with the xylem monoterpene profiles [28]; however the lodgepole pine and jack pine genes involved in the biosynthesis of monoterpenes are not known.

TPSs catalyse the ionization, rearrangement, and often the cyclization of short-chain isoprenyl diphosphates to produce the core structures of thousands of different terpenes found in plants [29-31]. Conifer mono-TPSs use geranyl diphosphate (GPP) as a substrate to form, typically in a stereo-specific fashion, acyclic, monocyclic or bicyclic monoterpenes. Mono-TPSs can be single- or, more commonly, multi-product enzymes [11,32]. Genomes of conifers harbour families of closely related mono-TPSs as was evident from EST analyses and full-length (FL)cDNA characterization of TPSs from Norway spruce (*Picea abies*), Sitka spruce (*P. sitchensis*) and white spruce (*P. glauca*) [32,33]. For example, analysis of ESTs from Sitka spruce and white spruce led to the identification and functional characterization of 15 mono-TPSs [32]. In contrast, for species of pine, to the best of our knowledge only three loblolly pine (*P. taeda*) mono-TPSs have been functionally characterized, which form either (+)-α-pinene, (−)-α-pinene, or (−)-α-terpineol as major products [34].

We report here the generation of large transcriptome sequence resources for jack pine and lodgepole pine obtained with Sanger, 454 and Illumina sequencing technologies. The quality of assemblies for FLcDNA discovery was evaluated by comparison with a core set of eukaryotic genes and by mining for sequences involved in terpenoid biosynthesis, specifically prenyl transferases (PTs), TPSs, and cytochrome P450s of the CYP720B subfamily. These enzymes control chain length (PT; [35]), core structures (TPSs, [11), and oxidative modifications (CYP720B; [36]) of terpenoids. This analysis led to the FLcDNA cloning and functional characterization of nine jack pine and eight lodgepole pine mono-TPSs. Monoterpene metabolite profiling of six tissue types from jack pine and lodgepole pine confirmed that we identified a set of mono-TPSs that account for the majority of monoterpenes in these tree species.

## Results

### Generation of jack pine and lodgepole pine transcriptome sequences and assemblies

We used the Sanger, 454 Titanium, and Illumina GA or Illumina HiSeq2000 platforms to generate transcriptome sequences for lodgepole pine and jack pine (Additional file 1: Table S1). For Sanger sequencing, we used normalized cDNA libraries from wound-treated stem tissues of a single lodgepole pine tree and four jack pine trees. Sequencing of the cDNA resulted in 41,134 paired-end reads for lodgepole pine and 36,334 paired-end reads for jack pine. For 454 and Illumina sequencing, libraries were made from the stem tissues of an individual 2-year old lodgepole pine and an individual 2-year old jack pine, both of which were wound- and methyl jasmonate-treated to induce defence responses [37]. 454 Titanium sequencing resulted in approximately 1.3 and 1.4 million reads for lodgepole pine and jack pine, respectively. Sequencing of the lodgepole pine cDNA library using the Illumina GAII platform yielded 58.5 million paired-end reads, whereas sequencing of jack pine cDNA using Illumina HiSeq2000 technology yielded 202.3 million paired-end reads.

Sanger sequences were CAP3 assembled [38]. The average insert sizes for the four normalized cDNA libraries varied from 929 to 1,136 bp. The lodgepole pine and jack pine assemblies each contained approximately 10,000 contigs and 4,000 singletons for 14,000 putatively unique genes (Additional file 2: Table S2). Newbler (Roche) assembly of 454 sequences gave 36,923 contigs for lodgepole pine and 33,974 contigs for jack pine, which corresponded to approximately 30,000 putatively unique genes for each species. Trinity [39] assembly of Illumina sequences gave 41,567 contigs for lodgepole pine and 55,416 contigs for jack pine, which also corresponded to approximately 30,000 putatively unique genes for each species. Assemblies of Sanger and 454 sequences, i.e. hybrid assemblies, were constructed using Newbler. These assemblies gave 33,589 and 31,327 contigs for lodgepole pine and jack pine, respectively, which represented approximately 20,000 putatively unique genes for each species (Additional file 2: Table S2).

### Assessment of assemblies for FLcDNA recovery using CEGMA genes

To evaluate and compare the assemblies for FLcDNA recovery, each assembly was first assessed for the presence of highly conserved eukaryotic proteins using “Core Eukaryotic Genes Mapping Approaches” (CEGMA) trained on a set of 458 *Arabidopsis thaliana* core proteins [40]. In the lodgepole pine and jack pine Sanger sequence assemblies 45-46% of the CEGMA protein matches were FL and an additional 37% were present but not FL (Additional file 3: Table S3). Forty-four of 84 CEGMA proteins that were not found in the jack pine assembly were also absent in the lodgepole pine assembly. The lodgepole pine and jack pine 454 sequence assemblies had almost perfect coverage with 97-98% of the CEGMA proteins represented, of which 70-71% were FL. Nine of the 11 proteins that were not detected in the lodgepole pine assembly were also not present in the jack pine assembly. Similarly, the lodgepole pine and jack pine Illumina sequence assemblies contained near perfect coverage (98-99%) of CEGMA proteins, with a slightly higher percentage of FL coverage (76.5% and 77.4%, respectively). Five of the six CEGMA proteins that were absent in the lodgepole pine Illumina assembly were also absent in the jack pine assembly. The hybrid Sanger/454 assemblies for lodgepole pine and jack pine each contained 97% of the CEGMA proteins, with approximately 70% of these being FL. Most CEGMA genes identified in the Sanger sequences were also present in the 454 and Illumina sequences. With one exception, all of the CEGMA genes that were missing in the Illumina sequence assemblies were also absent in the Sanger and 454 sequence assemblies.

### Assessment of assemblies for FLcDNA recovery using terpenoid pathway genes

To assess and compare the utility of the lodgepole pine and jack pine transcriptome assemblies for FLcDNA discovery of terpenoid pathway genes, each assembly was queried with known PTs, TPSs, and CYP450s from other species. These searches identified sequences for 7 unique PTs, 19 unique TPSs, and 8 unique CYP720Bs in lodgepole pine (total 34 unique genes); and 9 unique PTs, 21 unique TPSs and 8 unique CYP720Bs in jack pine (total 38 unique genes). We then assessed the coverage of these 34 and 38 genes with quality terms “full-length (FL)”, “not full-length (not FL)” or “not present (NP)” in each of the different assemblies (Additional file 4: Table S4). The 454 and 454/Sanger sequence assemblies had the best FL coverage with, respectively, 14 and 13 out of 34 targets being FL for lodgepole pine, and 20 out of 38 targets being FL for jack pine. The Illumina sequence assemblies had the highest overall coverage with 27 out of 34 genes present for lodgepole pine, and 32 out of 38 genes in jack pine, but these assemblies had lower FL sequence coverage. The Sanger sequence assemblies contained less than half of the target genes for each species, with no FL coverage for TPSs and CYP720Bs for lodgepole pine and only one FL TPS and one FL CYP720B for jack pine. Based on these results we used the Sanger/454 hybrid assembly for obtaining FLcDNAs of PTs, TPSs and CYP720Bs. The Illumina sequences will be useful for the discovery of additional genes of these families, which will require additional efforts to produce FLcDNAs.

### Mining of the 454/Sanger hybrid assembly for PT, TPS, and CYP720 genes

Overall, the lodgepole pine Sanger/454 hybrid assembly contained 259 CYP450-like contigs with 14 contigs representing the CYP720B subfamily. The jack pine assembly contained 339 CYP450-like contigs including 31 CYP720B contigs. As detailed above, analysis of the lodgepole pine and jack pine assemblies revealed, respectively, 6 and 7 unique CYP720B putative FL sequences. BLAST search outputs for PT and TPS sequences suggested that many of these genes were present in the assemblies with several closely related variants. Such closely related variants, which are typical for gene families of secondary metabolism, are extremely difficult to resolve in sequence databases. To further assess closely related variants, all sequences identified as PTs or TPSs were isolated from the larger sequence datasets and re-assembled using Phrap [41] to obtain an improved set of PT and TPS candidates. Mining of the lodgepole pine Phrap assembly identified 20 PT and 62 TPS sequences. Mining of the jack pine Phrap assembly identified 27 PT and 76 TPS candidates. Further manual inspection of the TPS candidates to remove sequences with mis-spliced introns or obvious sequence problems revealed a set of 10 FL and 23 partial TPS sequences in lodgepole pine (33 total), and 7 FL and 38 partial candidate TPSs in jack pine (45 total). Analysis of the PT sequences identified 7 FL and 6 partial sequences from lodgepole pine, and 9 FL and 2 partial sequences from jack pine.

### Cloning and characterization of mono-TPS FL cDNAs

Within the set of 33 lodgepole pine TPSs and 45 jack pine TPSs we searched for the subset of mono-TPSs using BLAST searches and expert sequence assessments against previously known conifer mono-TPSs, including those described in Keeling et al. [32]. Characterization of di-TPSs was recently published [42]. We found 7 FL and 10 partial mono-TPS candidates for lodgepole pine, and 5 FL and 24 partial mono-TPS candidates for jack pine. FL mono-TPSs were recovered from plasmids used for Sanger sequencing or by PCR using cDNA template. Rapid amplification of cDNA ends (RACE) led to the cloning of additional FL mono-TPSs. We cloned a total of 9 lodgepole pine and 11 jack pine FL mono-TPS cDNAs for recombinant expression in *E. coli* and characterization of TPS enzyme functions. Eight lodgepole pine and 9 jack pine TPS proteins showed activity with GPP as a substrate, confirming their identity as mono-TPSs. Assays using neryl diphosphate as a substrate produced only trace amounts of monoterpene products, and no product formation was detected with farnesyl diphosphate (FPP) or geranylgeranyl diphosphate (GGPP).

### Functional characterization of (−)- and (+)-α-pinene, (−)-β-phellandrene, and (−)-camphene synthases

**Figure 1:**
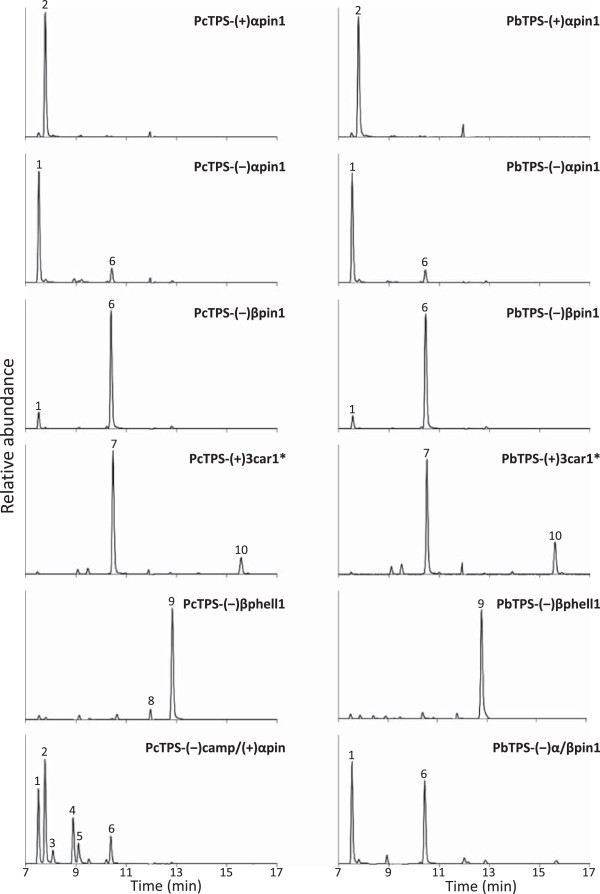
**Representative chiral gas chromatographic separation of enzymatic products from select recombinant lodgepole pine and jack pine monoterpene synthases that produce non-oxygenated monoterpenes as their main products.** Products representing greater than 5% of the total amount are labeled. 1, (−)-α-pinene; 2, (+)-α-pinene; 3, tricyclene; 4, (−)-camphene; 5, myrcene; 6, (−)-β-pinene; 7, (+)-3-carene; 8, (−)-α-phellandrene; 9, (−)-β-phellandrene; 10, terpinolene. *GC/MS traces for PcTPS-(+)3car1, PbTPS-(+)3car1 and PbTPS-(+)3car2 also showed 5%, 12% and 7% α-terpineol respectively, which elutes later than the scale shown, but are detailed in Additional file 5: Table S5.

Mono-TPS enzymes that produced product profiles dominated by 92% and 88% (+)-α-pinene were cloned from lodgepole pine [PcTPS-(+)αpin1] and jack pine [PbTPS-(+)αpin1], respectively (Figure 1, Additional file 5: Table S5). These proteins had 98% amino acid sequence identity to each other and to the previously characterized loblolly pine (+)-α-pinene synthase [34]. Two additional jack pine candidates (PbTPS-mono1, PbTPS-mono2) had 98% sequence identity to each other and 90% identity to the (+)-α-pinene synthases; however, these proteins showed no activity with GPP, GGPP or FPP. One protein from each species [PcTPS-(−)αpin1, PbTPS-(−)αpin1] produced 77-78% (−)-α-pinene and 10% (−)-β-pinene. These proteins also had 98% amino acid sequence identity to each other and to loblolly pine (−)-α-pinene synthase [34].

Two lodgepole pine cDNAs [PcTPS-(−)βphell1, PcTPS-(−)βphell2] and one jack pine cDNA [PbTPS-(−)βphell1] had 95-99% protein sequence identity to each other and had 70% sequence identity to PbTPS-(+)αpin1, PcTPS-(+)αpin1and PtTPS-(+)αpin1. These enzymes produced 82-88% (−)-β-phellandrene as the dominant product (Figure 1, Additional file 5: Table S5). A third lodgepole pine candidate [PcTPS-(−)camp/(+)αpin] had 95% identity to both the jack pine and lodgepole pine (−)-β-phellandrene synthases, and 70% identity to the (+)-α-pinene synthases, but produced 29% (−)-camphene and 26% (+)-α-pinene along with other minor products (Figure 1, Additional file 5: Table S5).

### Functional characterization of (−)-β-pinene, α-terpineol, and (+)-3-carene synthases

Two lodgepole pine mono-TPS candidates [PcTPS-(−)βpin1, PcTPS-mono1] and two jack pine candidates [PbTPS-(−)βpin1, PbTPS-(−)βpin2] had 96-98% amino acid sequence identity to each other and 91-93% sequence identity to loblolly pine (−)-α-terpineol synthase [34]. Surprisingly, none of these four proteins produced α-terpineol but instead produced 75-81% (−)-β-pinene and 8–13% (−)-α-pinene (Figure 1, Additional file 5: Table S5). The second lodgepole pine candidate (PcTPS-mono1) did not show any activity with GPP, FPP or GGPP, either as FL or truncated protein lacking the putative plastid targeting sequence. A third jack pine protein [PbTPS-(−)α/βpin1] had 92-97% sequence identity to the (−)-β-pinene synthases but instead produced a mixture of 39% (−)-α-pinene and 33% (−)-β-pinene.

**Figure 2:**
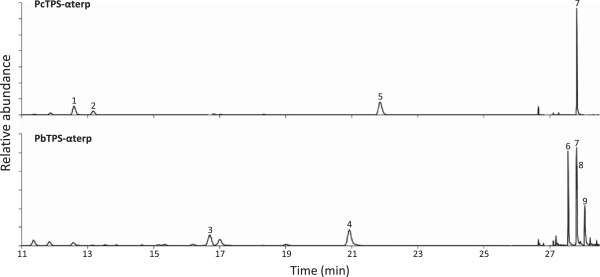
**Representative chiral gas chromatographic separation of enzymatic products from recombinant lodgepole pine and jack pine monoterpene synthases that produce oxygenated monoterpenes as their main product.** Products representing greater than 5% of the total amount are labeled. 1, myrcene; 2, (−)-sabinene; 3, (−)-limonene; 4, terpinolene; 5 1,8-cineole; 6, terpin-4-ol; 7, (−)-α-terpineol; 8, (+)-α-terpineol; 9, geraniol.

One candidate from each of jack pine (PbTPS-αterp) and lodgepole pine (PcTPS-αterp) had 92% sequence identity to each other and formed α-terpineol as the major product. These proteins had only 62% sequence identity to the PtTPS-(−)αterp [34] and were most closely related (77% identity) to 1,8-cineole synthases from white spruce and a white spruce hybrid [32]. Surprisingly, analysis of the stereochemistry of the α-terpineol product suggested that PbTPS-αterp produced a mixture of 44% (+) and 56% (−)-enantiomers, whereas PcTPS-αterp produced only the (−)-enantiomer. PbTPS-αterp also produced 17% terpin-4-ol, 10% geraniol, 9% terpinolene and 5% (−)-limonene, while PcTPS-αterp produced 32% 1,8-cineole, 9% (−)-sabinene and 8% myrcene as additional products (Figure 2, Additional file 5: Table S5).

Two jack pine candidates (PbTPS(+)3car1, PbTPS(+)3car1) and one lodgepole pine candidate (PcTPS(+)3car1) produced 56-68% (+)-3-carene and approximately 10% terpinolene (Figure 1, Additional file 5: Table S5). These proteins had 88–96% protein sequence identity to each other and were most closely related (69-79% identity) to (+)-3-carene synthases from Norway spruce, Sitka spruce and white spruce [32,43,44].

### Monoterpene profiles of jack pine and lodgepole pine

**Figure 3:**
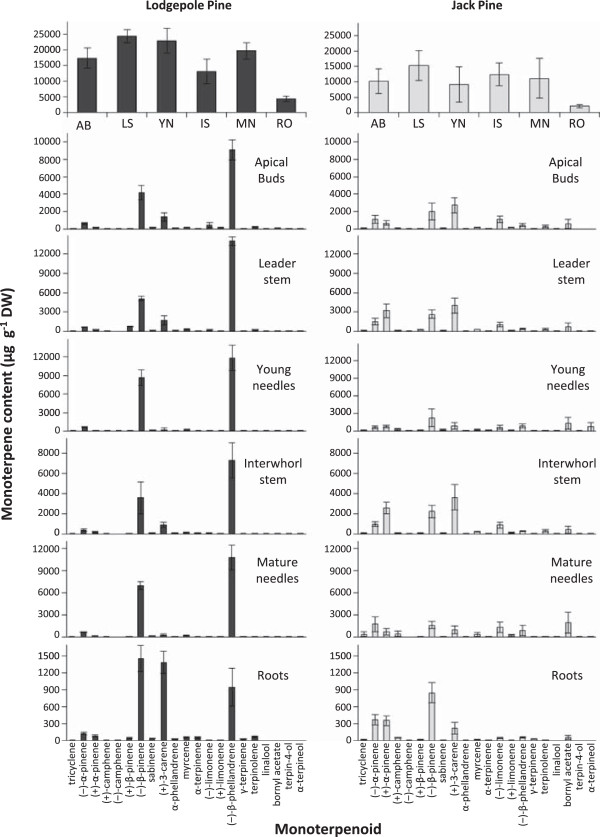
**Monoterpene profiles of six tissue/organ types from three-year old lodgepole pine and jack pine saplings.** The top left and right panels show total monoterpene contents for each tissue/organ type. All other panels show qualitative and quantitative details of individual monoterpenes for individual tissue/or organ types. Each bar represents the average ± standard error of 5 biological replicates with at least 2 technical replicates per sample. AB – apical buds, LS – leader stem, YN –needles from leader; IS – 1st interwhorl stem; MN – mature needles from 1st interwhorl; RO – roots.

To assess if products of the recombinant mono-TPS proteins were present in pine tissues, monoterpenes were extracted and analysed from the apical buds, leader stem, young needles (from the leader), first interwhorl stem, mature needles (from the first interwhorl) and roots of 3-year old jack pine and lodgepole pine saplings. In both pine species, the roots contained the lowest amount of total monoterpenes (Figure 3). Five of the six tissues of lodgepole pine contained 52-58% (−)-β-phellandrene and 20-35% (−)-β-pinene as the two most abundant monoterpenes, with the exception that lodgepole pine roots contained 21% (−)-β-phellandrene, 33% (−)-β-pinene and 32% (+)-3-carene. All lodgepole pine tissues contained approximately 3% (−)-α-pinene and the lodgepole pine apical buds, interwhorl stem and leader stem contained 7-8% (+)-3-carene and less than 1% of several additional monoterpenes including (+)-α-pinene, (+)-β-pinene, myrcene, terpinolene and both isomers of limonene and camphene (Figure 3).

Leader stem and interwhorl stem tissues of jack pine contained a complex mixture of monoterpenes, containing 26-29% (+)-3-carene, 21% (+)-α-pinene and 18% (−)-β-pinene, 8-10% (−)-α-pinene, 7% (−)-limonene, 4-5% bornyl acetate and 2% (−)-β-phellandrene, myrcene and terpinolene. Monoterpene profiles of jack pine needles contained 15-24% (−)-β-pinene, 14–18% bornyl acetate as well as 6-15% of (−)-α-pinene, (+)-α-pinene, (+)-3-carene, (−)-β-phellandrene, (−)-limonene. Jack pine apical buds contained 27% (+)-3-carene, 20% (−)-β-pinene, 6% bornyl acetate, 7% (+)-α-pinene as well as 11% (−)-α-pinene and (−)-limonene (Figure 3).

### Phylogeny of the lodgepole pine and jack pine mono-TPSs

**Figure 4:**
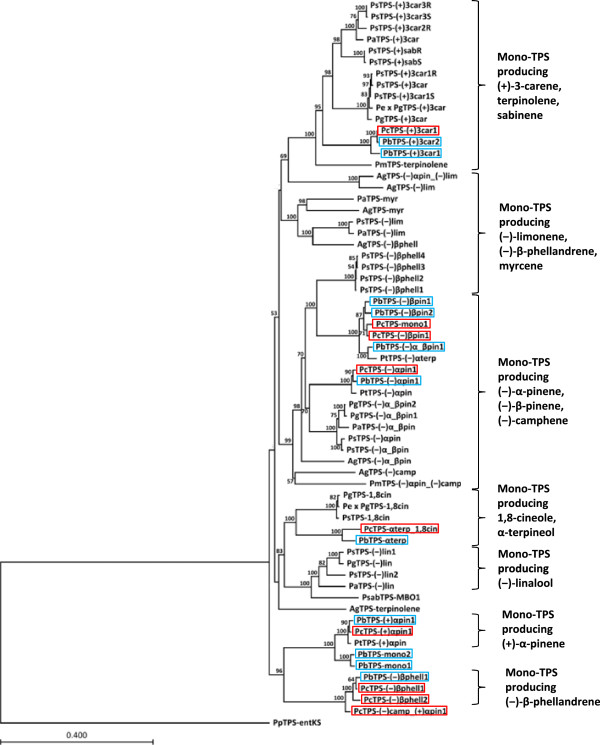
**Phylogeny of FL mono-TPSs from lodgepole pine and jack with previously characterized conifer mono-TPSs.** Bootstrap values greater than 50% are indicated at nodes. Abbreviations and NCBI accession numbers are located in Additional file 6: Table S6. Mono-TPSs characterized in this report are outlined in red (lodgepole pine) or blue (jack pine).

A neighbour joining phylogeny placed all of the FL jack pine and lodgepole pine mono-TPSs within the TPS-d1 family ([32]; Figure 4, Additional file 6: Table S6). Many of the jack pine and lodgepole pine mono-TPSs grouped together with functionally related mono-TPSs from other conifer species. For example, the jack pine and lodgepole pine (+)-3-carene synthases grouped together with spruce (+)-3-carene synthases [32,43,44] and the *Pseudotsuga menziesii* terpinolene synthase [45]. Similarly, the pine (−)-α-pinene synthases (Figure 1; Additional file 6: Table S6; [34]) grouped most closely with spruce and fir enzymes that produce (−)-α-pinene and (−)-β-pinene [32,33,46]. Conversely, the lodgepole pine and jack pine (−)-β-pinene synthases grouped together with the loblolly pine (−)-α-terpineol synthase [34] and were most closely related to the spruce (−)-β-phellandrene synthases [32], whereas the jack pine and lodgepole pine α-terpineol synthases grouped together with spruce enzymes that produce (−)-linalool and 1,8-cineole (Figure 2, [32,33]). The jack pine and lodgepole pine (−)-β-phellandrene synthases grouped together with pine (+)-α-pinene synthases (Figure 1; Additional file 1: Table S1; [34]) and grouped separately from other previously characterized conifer (−)-β-phellandrene synthases [32].

## Discussion

### Identification of FL transcripts of genes of terpenoid metabolism

We present transcriptome sequence resources generated with Sanger, 454, and Illumina technologies to enable gene discovery in jack pine and lodgepole pine. Different assemblies were generated to assess the ability to reconstruct FL transcripts. Previous work identified Newbler as ideal for obtaining FL transcripts from 454 sequences of non-model organisms [47]. We also used the Trinity assembler to process Illumina sequences. tBLASTn searches of the assemblies using CEGMA [40] proved useful as a general assessment of FL transcript recovery. The shorter length of the Illumina sequences did not appear to be a disadvantage and appeared to be compensated by the depth of coverage, which allowed us to recover most of the CEGMA genes with FL transcripts in both pine species.

A major challenge of conifer transcriptome analysis is the proper assembly of FL sequences of members of multigene families, where closely related members have divergent functions, and which are characteristic of secondary metabolism. TPS and P450 genes such as the conifer TPS-d and CYP720B genes are examples of such multigene families [31,32,36]. As a strategy for discovery and resolution of members of large gene families such as the TPSs in conifers we used the re-assembly of target genes after initial large-scale *de novo* transcriptome assembly. We have previously used this approach as part of a transcriptome assembly and gene discovery pipeline that was validated with known conifer di-TPSs and led to the successful discovery of new di-TPS genes of previously unknown functions in balsam fir (*Abies balsamea*) [48], lodgepole pine, and jack pine [42].

### Mono-TPSs accounted for major components of the monoterpene metabolite profiles of lodgepole pine and jack pine

The dominant monoterpenes across the different organ and tissue types of lodgepole pine were (−)-β-phellandrene and (−)-β-pinene (Figure 3). Consistent with these profiles, two (−)-β-phellandrene synthases and one (−)-β-pinene synthase were characterized from lodgepole pine. Additional mono-TPSs were also characterized from lodgepole pine that produced (+)-α-pinene, (−)-α-pinene, (−)-camphene, (+)-3-carene, and α-terpineol. The most abundant monoterpenes in jack pine were (+)-α-pinene, (−)-α-pinene, (−)-β-pinene and (+)-3-carene. We identified the mono-TPSs responsible for the biosynthesis of these metabolites as well as the synthases that produce (−)-β-phellandrene and α-terpineol. Bornyl acetate and (−)-limonene were also present in moderate amounts in all jack pine tissues tested. However, the synthases responsible for the production of these compounds have not been identified. In other conifers, mono-TPSs that produce (−)-limonene have been identified from grand fir [46], Norway spruce [33] and Sitka spruce [49]. Crude enzyme extracts from liverwort (*Conocephalum conicum*) catalyse the conversion of GPP to bornyl diphosphate [50], the likely precursor to bornyl acetate. A gene responsible for the biosynthesis of (+)-bornyl diphosphate was characterized in *Salvia officinalis*[51]. However, the genes involved in bornyl acetate biosynthesis have not been identified in any conifer species. The sequences of the TPSs that produce the remainder of the monoterpenes detected in lodgepole pine and jack pine, including (−)-limonene and bornyl acetate, are likely to be present within our assemblies in the form of partial transcripts. Additional approaches, such as targeted RACE, may be required to obtain the FL cDNAs for functional characterization. Among the partial or uncharacterized mono-TPS-like genes from jack pine and lodgepole pine, there may also be TPSs that use dimethylallyl diphosphate as a substrate to produce hemiterpenes, as was recently shown in *Pinus sabiniana*[52]. This possibility will be tested in future work. Using lodgepole pine and jack pine transcriptome resources, the discovery of a set of bifunctional and monofunctional di-TPSs was recently reported [42]. Together, these di-TPSs and the mono-TPSs described here account for the majority of oleoresin defence compounds of lodgepole pine and jack pine.

The discovery of two (+)-3-carene synthases from jack pine and one (+)-3-carene synthase from lodgepole pine may suggest that copy number variation in the (+)-3-carene synthases account for the difference in (+)-3-carene content in these trees, similar to the copy number variation of (+)-3-carene phenotypes in Sitka spruce [44]. Previous research has identified (+)-3-carene as important in the defence of Chinese pine (*Pinus tabuliformis*) against the red turpentine beetle (*Dendroctonus valens*) and its associated fungus (*Leptographium procerum*) [53]. Additionally, (+)-3-carene is associated with the resistance of Sitka spruce to the white pine weevil [44,54]. The (+)-3-carene content in all tissue types of jack pine was higher than the content of the corresponding tissue in lodgepole pine, consistent with previous comparisons of jack pine and lodgepole pine volatile emission and oleoresin content [27]. The monoterpene content in lodgepole pine, including (+)-3-carene, has strong genetic [55] and geographical [25] components, and has important consequences in MPB host colonization [14].

### Functional diversification of jack pine and lodgepole pine mono-TPSs

Gene duplication followed by sub- and neo-functionalization is likely the origin of the large conifer TPS gene family of oleoresin biosynthesis [31-33,56]. The 17 mono-TPSs characterized in this study provide additional insight into the molecular underpinnings of the monoterpene diversity observed across conifer species. The jack pine and lodgepole pine mono-TPS genes are members of the TPS-d1 family containing conifer mono-TPSs [31-33]. Many of the pine monoTPSs, including the genes responsible for (+)-3-carene and (−)-α-pinene biosynthesis, group phylogenetically with functionally similar mono-TPSs from loblolly pine, grand fir and spruce. This functional conservation across species suggests that considerable gene duplication and functionalization occurred prior to the speciation of pine, fir and spruce.

The jack pine and lodgepole pine (+)-α-pinene synthases and (−)-β-phellandrene synthases grouped together with the previously characterized loblolly pine (+)-α-pinene synthase [34] as a unique and apparently *Pinus* specific subclade within the TPS-d family (Figure 4). The jack pine and lodgepole pine (−)-β-phellandrene synthases grouped separately from the Sitka spruce [32] and grand fir (−)-β-phellandrene synthases [57] highlighting the multiple origins of (−)-β-phellandrene biosynthesis in conifers. Genes that produce (+)-α-pinene as their major product have not been identified in any conifer genus other than *Pinus*, suggesting this function may have evolved in the pine lineage after the separation from spruces and firs.

Three proteins from jack pine and lodgepole pine shared 91-93% sequence identity with the previously characterized loblolly pine (−)-α-terpineol synthase. Based on sequence identity, one may have predicted that the jack pine and lodgepole pine proteins would similarly produce α-terpineol. Surprisingly, these proteins produced 75-81% (−)-β-pinene and no α-terpineol. Previous reports demonstrate that a few amino acid substitutions are sufficient to alter the product profiles of mono-TPSs from grand fir [58,59]. The high level of sequence identity between these functionally distinct proteins from jack pine, lodgepole pine and loblolly pine serves as an example of the functional plasticity observed in conifer mono-TPS.

## Conclusions

Here, we present large transcriptome resources for lodgepole pine and jack pine generated on Sanger, 454 and Illumina sequencing platforms. Beyond using highly conserved plant and eukaryotic genes for quality assessment of assemblies, we successfully focused on FLcDNA recovery and resolution of closely related sequences characteristic of large gene families of conifer defence and secondary metabolism. Expert-curated assemblies and annotation identified a substantial number of terpenoid pathway genes in the two pine species investigated here. The closely related jack pine and lodgepole pine have unique monoterpene profiles that function as defences and semiochemicals in the interactions with MPB and MPB-associated fungi as well as other pests. The diverse and different functions of the trees’ mono-TPSs identified here account for many of the major and minor monoterpenes in different tissues, yet also point to enzymes of terpene biosynthesis which remain to be discovered. The genes identified provide a foundation to further investigate the role of these compounds as well their intra- and interspecific variations and dynamics in the defence of jack pine and lodgepole pine to the MPB.

## Methods

### Origin and treatment of pine tissues and RNA extractions

For Sanger sequencing, six-year old lodgepole pine (*Pinus contorta*) trees were provided by the BC Ministry of Forestry and Range. Trees were maintained in pots, outside at the University of British Columbia Vancouver campus. One-year old jack pine (*Pinus banksiana*) from the “Prince Albert West” seedlot were obtained from Forest First, SK and maintained in a growth chamber at the University of Alberta. The leader and top three interwhorls of a single lodgepole pine tree and the top interwhorl from four jack pine trees were mechanically wounded with a razor blade 1, 2, 4, and 8 days prior to harvesting the tissue. At harvest, the stem sections were cut from the tree and the bark manually separated from the xylem, and the needles removed. Bark and xylem were immediately frozen separately in liquid nitrogen and stored at −80°C.

For 454 and Illumina sequencing and metabolite analysis, jack pine (clone ID PSB 410 1 + 0) and lodgepole pine (clone ID PLI 144) saplings were maintained outdoors at the University of British Columbia. For RNA extraction, four two-year old saplings of each species were moved into the greenhouse and were maintained at 24°C and 16 h light per day for two weeks prior to induction of defence gene expression. The stem of each tree was wounded along the whole stem with a razor blade incision 2 – 3 cm apart, and the aerial portion of the tree was sprayed with 50 ml of 0.1% methyl jasmonate. One quarter of the stem (combined bark and xylem) of each tree was harvested 2 h, 6 h, 24 h and 48 h following treatment and the four time points from each individual were pooled prior to RNA extraction. For metabolite profiling, five three-year old saplings of each species were moved into the greenhouse for two weeks prior to harvest. Tissue samples were harvested from each tree, and flash frozen and stored at −80°C until processing with the exception that combined bark and xylem tissue was cut into 1 cm sections prior to freezing.

### RNA isolation

For Sanger sequencing, total RNA from the separated bark and xylem tissues was extracted from each of the four stem sections of the single lodgepole pine tree as described previously [60]. For jack pine, the total RNA was extracted from each individual bark and xylem tissue sample according to Pavy et al. [61] and pooled. For RACE, full-length cDNA cloning and template for 454 and Illumina sequencing, RNA from single jack pine and lodgepole pine trees was extracted and converted to cDNA as described previously [44].

### cDNA library construction and transcriptome sequencing

For Sanger cDNA library construction, mRNA was purified from total RNA using the Oligotex mRNA Kit (Qiagen; http://www.qiagen.com) and quantified by RiboGreen (Invitrogen; http://www.invitrogen.com). cDNA libraries were prepared using the Creator SMART cDNA Library Construction Kit (Clontech; http://www.clontech.com) and the Trimmer-Direct cDNA Normalization Kit (Evrogen; http://www.evrogen.com). First strand cDNA was prepared from 225–1,500 ng of total RNA in jack pine or 106–1,300 μg of poly(A)^+^ RNA in lodgepole pine, SuperScript III reverse transcriptase (Invitrogen), CDS-3 M primer (Evrogen), and the SMART IV Oligonucleotide (Clontech). Second strand cDNA was prepared by LD-PCR with Phusion Hot Start DNA Polymerase (Finnzymes, http://www.finnzymes.fi). cDNA was normalized with duplex-specific nuclease and amplified following the Trimmer-Direct protocol. The cDNA was then digested with *Sfi*I, size fractionated by gel filtration, and cDNA larger than approx. 500 bp was ligated into pDNR-LIB (Clontech). The ligations were then transformed into ElectroMAX DH10B T1 Phage-Resistant electro-competent cells (Invitrogen), titred and submitted to the Michael Smith Genome Sciences Centre for arraying and sequencing. Sanger sequencing (paired end reads) was completed using M13 forward and reverse primers. For lodgepole pine, 10,000 cDNA clones each were sequenced from the bark and xylem cDNA libraries. For jack pine, 15,550 and 6,528 cDNA clones were sequenced from the bark and xylem libraries, respectively.

For 454 and Illumina sequencing, cDNA libraries were prepared and sequenced at the McGill University and Génome Québec Innovation Centre, in Montreal, QC. For Roche-GS-FLX – Titanium (454) sequencing, 200 ng of mRNA was purified using the Dynabeads mRNA purification kit (Invitrogen) and was fragmented using a ZnCl_2_ buffer. cDNA libraries were prepared using the GS FLX Titanium Series cDNA Rapid Library preparation kit (Roche; http://www.roche.com) and were subjected to two half-plates (single end reads) of sequencing. For Illumina sequencing, the same lodgepole pine total RNA and a new sample of total RNA from the same jack pine individual were subjected to mRNA purification and cDNA library construction using the mRNA Seq Sample Preparation Kit (Illumina; http://www.illumina.com). The lodgepole pine library was subjected to 1 lane of sequencing (108 bp paired-end reads) using the Illumina Genome Analyzer iiX (Illumina GAII) platform, while the jack pine library was subjected to 1 lane of sequencing (100 bp paired-end reads) using the Illumina HiSeq 2000 platform.

### Filtering and assembly of jack pine and lodgepole pine transcriptome sequences

After removal of poor quality and chimeric reads, raw Sanger sequences were screened for contaminants against *Escherichia coli* K12, fungi, various bacterial and archaea genomes, and all *Insecta* ESTs. These sequences were then trimmed using Seqclean to remove any remaining adapter sequence. 454 and Illumina sequences were inspected visually using FastQC. A custom Perl script was developed to trim the adapter remnants in both cases with the fastq file for 454 and Illumina obtained from the sequencing centre. The Sanger sequences were assembled with CAP3 [38]. Newbler (version 2.6; Roche) was used in both 454 only and 454/Sanger hybrid assembly. Trinity (version 20110519; [39]) was used in the *de novo* assembly of Illumina GA/HiSeq sequences.

### Identification of tentatively unique genes, CEGMA FL proteins and comparison to FL terpenoid pathway targets

Arabidopsis CEGMA peptide sequences were used to query the 6-frame translated transcriptomic assemblies using TBLASTN with an e-value cut-off of 1 × 10^-20^. Similarly, MEGABLAST was used to query the assemblies for FL terpenoid biosynthetic pathway genes with a cut-off set at 99% identity. A custom Perl script was developed to assess the contiguity of the predicted transcripts. Only the top hit in the BLAST alignment, with at least 90% coverage of the query peptide was considered to be FL for CEGMA genes. Terpenoid biosynthetic pathway genes that were identified as FL in at least one assembly, or that had been obtained as FL by RACE, were BLAST searched against each assembly to compare the utility of these assemblies for defence gene discovery. A final set of 7 PT, 19 TPS and 8 CYP720B genes from lodgepole pine and 9 PT, 21 TPS and 8 CYP720B from jack pine were considered FL and used for this analysis. A more stringent 95% coverage was used to be considered FL for terpenoid biosynthetic genes.

### Identification of candidate TPSs for cDNA cloning

The lodgepole pine and jack pine 454 Newbler assemblies were BLAST searched with a set of 107 previously characterized mono-, sesqui- and di-TPSs, 15 previously characterized PTs and 468 previously characterized P450s. The reads contained in each of the contigs identified in the Newbler assembly that had an e-value of less than 1 × 10^-5^ to terpenoid biosynthetic pathway genes were then re-assembled using Phrap [62] to obtain a finalized list of candidate genes.

### Cloning and characterization of monoTPSs

FL clones were retrieved from the cDNA library used for Sanger sequencing and were cloned from pDNR-LIB into the pET28b(+) vector (EMD Chemicals, http://www.emd-chemicals.ca) for expression. For additional clones, if RACE was required, 1 μg of total RNA was processed using the SMARTer RACE cDNA amplification kit (Clontech) and this cDNA was used as a template with gene specific primers (Additional file 7: Table S7) and the universal primer mix as per the manufacturer’s protocol. For cloning of FL targets from the same jack pine and lodgepole pine individuals that had been subjected to high throughput sequencing, 50 ng of jack pine RNA and 90 ng of lodgepole pine RNA were converted to cDNA using the Superscript III First-Strand Synthesis System (Invitrogen) and used as a template with gene specific primers (Additional file 7: Table S7). cDNA clones were either cloned directly into the pET28b(+) vector using the In-Fusion PCR cloning system (Clontech) or were subcloned into pJET1.2 (Fermentas; http://www.fermentas.com) prior to subcloning to pET28b(+) using In-Fusion cloning. Sequencing confirmed that the FL sequences were in frame with an N-terminal 6 × His tag in pET28b(+). Recombinant proteins were expressed, Ni-affinity purified on His SpinTrap columns (GE Healthcare), and assayed in single vials as described previously [44,56,63]. Bacterial pellet extracts containing recombinant protein were assayed with GPP (20 μM), *E*,*E*- FPP (70 μM) and GGPP (40 μM) in the appropriate buffers [32] and the products were subjected to analysis by GC/MS. Candidate clones that showed activity with GPP were extracted, purified, and assayed in triplicate using GPP and its isomer NPP as substrates.

### Monoterpene extraction from jack pine and lodgepole pine

Approximately 0.1 g of each of six different tissue or organ types harvested from 3-year old jack pine and lodgepole pine saplings were extracted in 1.5 mL of *tert*-butyl methyl ether (Sigma) containing 1.2 mM isobutylbenzene as an internal standard. Following shaking overnight at room temperature, 1 ml of extract was transferred to a fresh vial and processed as described previously [54]. Extractions were repeated with five biological replicates and three technical replicates per tissue type. The six tissue types were: flushing apical buds, combined bark and xylem from the leader, young needles (from leader), combined bark and xylem from the first interwhorl, mature needles from the first interwhorl, and roots.

### GC/MS analysis of monoterpenes

Gas chromatography mass spectrometry (GC/MS) analysis was performed on an Agilent 6890A Series GC system coupled to an Agilent 5975 Inert XL mass spectrometer (70 eV), with an Agilent 7683 autosampler (Agilent Technologies, http://www.agilent.com) as described in the supplemental material of a previous publication [44]. All analyses were performed using pulsed splitless injection mode with an injector temperature of 250°C. Data was analysed using Enhanced MSD Chemstation E.01.00 (Agilent Technologies). Data was collected using both full scan (*m/z* 40–400) and monoterpene-specific selective ion-monitoring (*m/z* 69, 93, 121, 134, 136) mode. Monoterpenes were identified by comparison of mass spectra and retention times of authentic standards, and by comparison to mass spectral libraries (Wiley7Nist05). Response factors were calculated based on a known concentration of isobutylbenzene, and these values were used to quantify the monoterpene compounds.

For enzyme assays, monoterpenes were analyzed on a DB-Wax capillary column (J&W 122–7032; 250 μm internal diameter, 30 m length, 0.25 μm film thickness, initial flow 1.0 ml He min^-1^) starting at a temperature of 40°C which was held for 4 min. The temperature was increased by 3°C per min^-1^ to 85°C, at which point the temperature was increased by 30° min^-1^ to a final temperature of 250°C, which was held for 3 min (total run time 27 min).

Monoterpenes extracted from pine tissue samples were separated on a SGE SolGel-Wax capillary column (SGE Analytical Science 054796, 250 μm internal diameter, 30 m length, 0.25 μm film thickness, initial flow 1.1 ml He min^-1^). An initial temperature of 40°C was held for 4 min, at which point the temperature was increased at a rate of 3°C min^-1^ to 80°C and then by 40°C min^-1^ to a final temperature of 275°C, which was held for 5 min (total run time 27.21 min).

For stereochemical analysis of enzyme assay products and tissue extracts, monoterpenes were subjected to chiral separation on a Cylcodex B capillary column (J&W 112–2532; 250 μm internal diameter, 30 m length, 0.25 μm film thickness, initial flow 0.8 ml He min^-1^) starting at an initial temperature of 60°C, which was held for one minute. The temperature was increased at a rate of 1°C min^-1^ to 84°C, and then increased at a rate of 50°C min^-1^ to a final temperature of 240°C, which was held for 5 min (total run time of 33.12 min).

### Phylogenetic analysis

CLC Main Workbench, Version 6.2 (CLCbio; http://www.clcbio.com) was used for all sequence analyses including alignments and phylogenetic analyses. Phylogenetic analysis and calculation of bootstrap values (100 replicates) was executed using the neighbour-joining algorithm with the manufacturer’s settings following alignment with the CLC bio MUSCLE plug-in.

## Abbreviations

CEGMA: Core Eukaryotic Genes Mapping Approach; di-TPS: Diterpene synthase; FL: Full-length; FPP: Farnesyl diphosphate; GC/MS: Gas chromatography mass spectrometry; GGPP: Geranylgeranyl diphosphate; GPP: Geranyl diphosphate; Mono-TPS: Monoterpene synthase; MPB: Mountain pine beetle; NPP: Neryl diphosphate; P450: Cytochrome P450; PT: Prenyl transferase; TPS: Terpene synthase.

## Competing interests

The authors declare that they have no competing interests.

## Authors’ contributions

DEH, MMSY, SJ, ALQ, HKD, ML, HH, CIK performed experiments and data analysis. AA-V, JEKC provided new materials. NYL, TRD, SKC, SJMJ provided bioinformatics support. CB, JB conceived the study. DEH, MY, JB analysed results and wrote the paper. All authors read and approved the final manuscript.

## Supplementary Material

Additional file 1: Table S1 Development of transcriptome sequence resources for lodgepole pine and jack pine. Click here for file

Additional file 2: Table S2 Assembly of transcriptome sequences for lodgepole pine and jack pine. Click here for file

Additional file 3: Table S3 Assessment of different sequencing platforms and assembly qualities using Core Eukaryotic Genes Mapping Approaches (CEGMA). Click here for file

Additional file 4: Table S4 Assessment of assembly quality of (A) lodgepole pine and (B) jack pine transcriptome data for presence of full length (FL) terpenoid pathway genes. Click here for file

Additional file 5: Table S5 Functional characterization of monoterpene synthases from A) lodgepole pine and B) jack pine. Click here for file

Additional file 6: Table S6 NCBI Accession numbers of the amino acid sequences used for phylogenetic analysis of functionally characterized conifer monoterpene synthases. Click here for file

Additional file 7: Table S7 Gene specific primers. Click here for file

## Database submissions

Sequence data from this article have been deposited in NCBI with the following accession numbers. Sanger ESTs: NCBI dbEST GT229582–GT270713 (lodgepole pine) and GW738148-GW774480 (Jack pine); 454 and Illumina ESTs: NCBI SRA BioProject SRP009894; FL mono-TPS: NCBI GenBank accession numbers: JQ240290-JQ240309. The Tria Project is represented by NCBI umbrella BioProject PRJNA169907.
