# Supplementary material for: Transcriptome resources and functional characterization of monoterpene synthases for two host species of the mountain pine beetle, lodgepole pine (Pinus contorta) and jack pine (Pinus banksiana)
Source: BMC Plant Biol. 2013 May 16;13:80. doi: 10.1186/1471-2229-13-80 (PMC3668260; doi:10.1186/1471-2229-13-80)
Supplement: Additional file 1: Table S1 — Development of transcriptome sequence resources for lodgepole pine and jack pine. [file 1471-2229-13-80-S1.pdf]

Table S1. Development of transcriptome sequence resources for lodgepole pine and jack pine. cDNA was sequenced using three different sequencing platforms i) Sanger sequencing with paired end reads, ii) Roche 454 Titanium sequencing iii) Illumina GA (lodgepole pine; 108 bp paired-end reads) or Illumina HiSeq2000 (jack pine; 100 bp paired-end reads).

Species	Sequencing platform	Quantity of sequencing	# Reads	# bp
Lodgepole pine	Sanger	22,000 cDNA clones	41,134	31,362,218
	454 Titanium	2 x half plate	1,280,650	522,187,905
	Illumina GA	1 lane	58,480,990	6,315,946,920
Jack pine	Sanger	20,000 cDNA clones	36,334	27,748,542
	454 Titanium	2 x half plate	1,397,993	536,550,760
	Illumina HiSeq2000	1 lane	202,383,402	20,238,340,200
