# Supplementary material for: Transcriptome resources and functional characterization of monoterpene synthases for two host species of the mountain pine beetle, lodgepole pine (Pinus contorta) and jack pine (Pinus banksiana)
Source: BMC Plant Biol. 2013 May 16;13:80. doi: 10.1186/1471-2229-13-80 (PMC3668260; doi:10.1186/1471-2229-13-80)
Supplement: Additional file 2: Table S2 — Assembly of transcriptome sequences for lodgepole pine and jack pine. [file 1471-2229-13-80-S2.pdf]

Table S2. Assembly of transcriptome sequences for lodgepole pine and jack pine. Sequences were assembled individually by platform, or as hybrid assemblies, and assessed for the number of contigs, singletons and putatively unique genes.

	Sequence source	Assembler	# Contigs[1,2]	# Singletons[1,2]	# Putatively Unique Genes[1,2]
Lodgepole pine	Sanger	CAP3	9,804	4,039	13,843
	454 Titanium[1]	Newbler	36,923	73,343	30,262
	Illumina GA[2]	Trinity	41,567	N/A	30,748
	Sanger + 454 Hybrid[1]	Newbler	33,589	116,424	21,362
Jack pine	Sanger	CAP3	9,950	3,762	13,712
	454 Titanium[1]	Newbler	33,974	68,790	27,639
	Illumina HiSeq2000[2]	Trinity	55,416	N/A	34,180
	Sanger + 454 Hybrid[1]	Newbler	31,327	114,303	20,169

[1] Contig counts in the context of the 454 assembly represent the number of isotigs greater than or equal to 500 bp. Singleton counts are calculated by reads labelled as 'Singleton' in the file 454ReadStatus.txt. Tentatively unique gene count is calculated by the number of unique isogroups containing isotigs greater than or equal to 500 bp.

[2] Contig counts in the context of the Illumina assembly represent the number of contigs greater than or equal to 500 bp. The Trinity assembler does not keep track of which sequences are being used in the assembly, thus the number of singletons is not available (N/A). Tentatively unique gene count is calculated by the unique component count in the result file Trinity.fasta.
