# Supplementary material for: Transcriptome resources and functional characterization of monoterpene synthases for two host species of the mountain pine beetle, lodgepole pine (Pinus contorta) and jack pine (Pinus banksiana)
Source: BMC Plant Biol. 2013 May 16;13:80. doi: 10.1186/1471-2229-13-80 (PMC3668260; doi:10.1186/1471-2229-13-80)
Supplement: Additional file 3: Table S3 — Assessment of different sequencing platforms and assembly qualities using Core Eukaryotic Genes Mapping Approaches (CEGMA). [file 1471-2229-13-80-S3.pdf]

Table S3. Assessment of different sequencing platforms and assembly qualities using Core Eukaryotic Genes Mapping Approaches (CEGMA). Individual and hybrid assemblies were assessed for the presence and full-length cDNA coverage of 458 core eukaryotic proteins using the minimum criteria of at least 90% coverage with a TBLASTN e-value cut-off of 1×10^{-20}.

Species	Assembly	# of CEGMA proteins	
		in assembly	putatively full length
Lodgepole pine	Sanger (CAP3)	377	216
	454 (Newbler)	447	330
	Illumina (Trinity)	452	358
	Sanger + 454 (Newbler)	444	325
Jack pine	Sanger (CAP3)	374	211
	454 (Newbler)	446	331
	Illumina (Trinity)	450	362
	Sanger + 454 (Newbler)	445	322
