# Supplementary material for: Transcriptome resources and functional characterization of monoterpene synthases for two host species of the mountain pine beetle, lodgepole pine (Pinus contorta) and jack pine (Pinus banksiana)
Source: BMC Plant Biol. 2013 May 16;13:80. doi: 10.1186/1471-2229-13-80 (PMC3668260; doi:10.1186/1471-2229-13-80)
Supplement: Additional file 4: Table S4 — Assessment of assembly quality of (A) lodgepole pine and (B) jack pine transcriptome data for presence of full length (FL) terpenoid pathway genes. [file 1471-2229-13-80-S4.pdf]

Table S4. Assessment of assembly quality of (A) lodgepole pine and (B) jack pine transcriptome data for presence of full length (FL) terpenoid pathway genes. Targets that had one contiguous sequence with coverage of at least 95 % length and with at least 99 % nucleotide sequence identity were considered to be full length (FL). Abbreviations: NP – targets not present; PT – prenyltransferases; TPS – terpene synthase; P450 – cytochrome P450.;

(A) Lodgepole pine

**Assembly**	**PT (7 unique genes)**			**TPS-d (19 unique genes)**			**P450 – CYP720B (8 unique genes)**		
	**FL**	**Not FL**	**NP**	**FL**	**Not FL**	**NP**	**FL**	**Not FL**	**NP**
Sanger (CAP3)	5	2	-	0	6	13	0	2	6
454 (Newbler)	4	2	1	6	3	10	6	1	1
Illumina (Trinity)	4	3	-	5	7	7	1	7	-
Sanger/454 Hybrid	6	1	-	3	11	5	6	0	2

(B) Jack pine

**Assembly**	**PT (9 unique genes)**			**TPS-d (21 unique genes)**			**P450 – CYP720B (8 unique genes)**		
	**FL**	**Not FL**	**NP**	**FL**	**Not FL**	**NP**	**FL**	**Not FL**	**NP**
Sanger (CAP3)	2	3	4	1	4	16	1	5	2
454 (Newbler)	7	1	1	7	4	10	6	1	1
Illumina (Trinity)	5	4	-	7	9	5	3	4	1
Sanger/454 Hybrid	7	2	-	6	6	9	7	1	-
