# Supplementary material for: Transcriptome resources and functional characterization of monoterpene synthases for two host species of the mountain pine beetle, lodgepole pine (Pinus contorta) and jack pine (Pinus banksiana)
Source: BMC Plant Biol. 2013 May 16;13:80. doi: 10.1186/1471-2229-13-80 (PMC3668260; doi:10.1186/1471-2229-13-80)
Supplement: Additional file 5: Table S5 — Functional characterization of monoterpene synthases from A) lodgepole pine and B) jack pine. [file 1471-2229-13-80-S5.pdf]

Table S5. Functional characterization of monoterpene synthases from A) lodgepole pine and B) jack pine. Products representing more than 5% of the total amount of monoterpenes produced by the enzyme are listed as percent of total product +/- SE.

A) Lodgepole pine

	% of total products												
	**tricyclene**	**(+)-α-pinene**	**(−)-α-pinene**	**(−)-camphene**	**(−)-β-pinene**	**(−)-sabinene**	**(+)-3-carene**	**(−)-α-phellandrene**	**myrcene**	**(−)-β-phellandrene**	**1,8-cineole**	**terpinolene**	**(−)-α terpineol**
**PcTPS-(+) αpin1**		**88.2 ± 0.48**											
**PcTPS-(−)αpin1**			**76.6 ± 2.2**		9.88 ± 1.8								
**PcTPS-(−)βpin1**			9.03 ± 0.05		**79.0 ± 0.16**								
**PcTPS-(+)3car1**							**67.6 ± 0.89**					12.5 ± 0.14	5.73 ± 0.42
**PcTPS-(−)βphell1**								5.14 ± 0.08		**87.5 ± 0.27**			
**PcTPS-(−)βphell2**										**81.8 ± 0.30**			
**PcTPS-(−)camp/ (+)αpin1**	6.78 ± 0.04	**26.2 ± 0.13**	17.9 ± 0.09	**29.1 ± 0.13**	7.89 ± 0.04				5.01 ± 0.04				
**PcTPS-αterp/1,8cin**						8.58 ± 0.20			8.06 ± 0.15		**32.4 ± 0.34**		**36.8 ± 0.20**

B) Jack pine

	% of total products										
	(+)-α-pinene	(−)-α-pinene	(−)-β-pinene	(+)-3-carene	(−)-limonene	(−)-β-phellandrene	terpinolene	terpin-4-ol	(+)-α terpineol	(-)-α terpineol	geraniol
**PbTPS-(+)αpin1**	**91.7 ± 0.48**										
**PbTPS-(−)αpin1**		**77.9 ± 0.17**	9.62 ± 0.15								
**PbTPS-(−)βpin1**		8.42 ± 0.16	**80.6 ± 0.22**								
**PbTPS-(−)βpin2**		13.1 ± 0.13	**75.2 ± 0.18**								
**PbTPS-(+)3car1**				**55.6 ± 3.5**			20.8 ± 1.4		5.30 ± 2.0	7.26 ± 2.8	
**PbTPS-(+)3car2**				**69.6 ± 0.31**			6.81 ± 0.23			6.86 ± 0.13	
**PbTPS-(−)βphell1**						**85.9 ± 0.14**					
**PbTPS-(−)α/βpin1**		**39.4 ± 0.99**	**33.5 ± 0.65**								
**PbTPS-αterp**					5.26 ± 0.13		9.2 ± 0.20	**17.3 ± 0.35**	**14.5 ± 0.64**	**18.1 ± 0.80**	**9.96 ± 0.74**
