# Supplementary material for: Transcriptome resources and functional characterization of monoterpene synthases for two host species of the mountain pine beetle, lodgepole pine (Pinus contorta) and jack pine (Pinus banksiana)
Source: BMC Plant Biol. 2013 May 16;13:80. doi: 10.1186/1471-2229-13-80 (PMC3668260; doi:10.1186/1471-2229-13-80)
Supplement: Additional file 6: Table S6 — NCBI Accession numbers of the amino acid sequences used for phylogenetic analysis of functionally characterized conifer monoterpene synthases. [file 1471-2229-13-80-S6.pdf]

Table S6. NCBI Accession numbers of the amino acid sequences used for phylogenetic analysis of functionally characterized conifer monoterpene synthases

Species	Abbreviation	Function	Accession #	Reference
Abies grandis (Ag)	AgTPS-myr	myrcene synthase	AAB71084	Bohlmann et al., 1997
	AgTPS-(–)-lim	(–)-limonene synthase	AAB70907	Bohlmann et al., 1997
	AgTPS-(–)α/βpin	(–)-α/β-pinene synthase	AAB71085	Bohlmann et al., 1997
	AgTPS-(–)camp	(–)-camphene synthase	AAB70707	Bohlmann et al., 1997
	AgTPS-(–)βphell	(–)-β-phellandrene synthase	AAF61453	Bohlmann et al., 1999
	AgTPS-terpinolene	terpinolene synthase	AAF61454	Bohlmann et al., 1999
	AgTPS-(–)αpin/(–)lim	(–)-α-pinene/(–)-limonene synthase	AAF61455	Bohlmann et al., 1999
Picea abies (Pa)	PaTPS-(+)3car	(+)-3-carene synthase	AAO73863	Fäldt et al., 2003
	PaTPS-(–)α/βpin	(–)-α/β-pinene synthase	AAS47692	Martin et al., 2004
	PaTPS-(–)lin	(–)-linalool synthase	AAS47693	Martin et al., 2004
	PaTPS-(–)lim	(–)-limonene synthase	AAS47694	Martin et al., 2004
	PaTPS-myr	myrcene synthase	AAS47696	Martin et al., 2004
Picea glauca (Pg)	PgTPS-(+)3car	(+)-3-carene synthase	ACM04452	Hamberger et al., 2009
	PgTPS-1,8cin	1,8-cineole synthase	ADZ45498	Keeling et al., 2011
	PgTPS-(–)lin	(–)-linalool synthase	ADZ45500	Keeling et al., 2011
	PgTPS-(–)α/βpin1	(–)-α/β-pinene synthase1	ADZ45507	Keeling et al., 2011
	PgTPS-(–)α/βpin2	(–)-α/β-pinene synthase2	ADZ45508	Keeling et al., 2011
Picea engelmannii x *Picea glauca* *(PexPg)*	Pe x PgTPS-1,8cin	1,8-cineole synthase	ADZ45497	Keeling et al., 2011
	Pe x PgTPS-(+)3car	(+)-3-carene synthase	ADZ45510	Keeling et al., 2011
Pinus contorta (Pc)	PcTPS-(+)αpin1	(–)-α-pinene synthase1	JQ240295	This study
	PcTPS-(–)αpin1	(–)-α-pinene synthase1	JQ240303	This study
	PcTPS-(–)βpin1	(–)-β-pinene synthase1	JQ240293	This study
	PcTPS-(+)3car1	(+)-3-carene synthase1	JQ240307	This study
	PcTPS-(–)camp/(+)αpin1	(–)-camphene/(+)-α-pinene synthase1	JQ240299	This study

	PcTPS-(−)βphell1	(−)-β-phellandrene synthase1	JQ240301	This study
	PcTPS-(−)βphell2	(−)-β-phellandrene synthase2	JQ240300	This study
	PcTPS-αterp/1,8cin	(+)-α-terpineol/1,8 cineole synthase	JQ240309	This study
	PcTPS-monoTPS1	monoterpene synthase like1	JQ240294	This study
Pinus banksiana (Pb)	PbTPS-(−)α/βpin1	(−)-α/β-pinene synthase1	JQ240290	This study
	PbTPS-(+)αpin1	(+)-α-pinene synthase1	JQ240298	This study
	PbTPS-(−)αpin1	(−)-α-pinene synthase1	JQ240304	This study
	PbTPS-(−)βpin1	(−)-β-pinene synthase1	JQ240291	This study
	PbTPS-(−)βpin2	(−)-β-pinene synthase2	JQ240292	This study
	PbTPS-(+)3car1	(+)-3-carene synthase1	JQ240305	This study
	PbTPS-(+)3car2	(+)-3-carene synthase2	JQ240306	This study
	PbTPS-(−)βphell1	(−)-β-phellandrene synthase1	JQ240302	This study
	PbTPS-αterp	(+)-α-terpineol synthase	JQ240308	This study
	PbTPS-mono1	monoterpene synthase like1	JQ240296	This study
	PbTPS-mono2	monoterpene synthase like1	JQ240297	This study
Pinus sabiana (Psab)	PsabTPS-MBO1	2-methyl-3-buten-2-ol synthase	AEB53064	Gray et al., 2011
Pinus taeda (Pt)	PtTPS-(−)αpin	(−)-α-pinene synthase	AAO61225	Phillips et al., 2003
	PtTPS-(−)αterp	α-terpineol synthase	AAO61227	Phillips et al., 2003
	PtTPS-(+)αpin	(+)-α-pinene synthase	AAO61228	Phillips et al., 2003
Picea sitchensis (Ps)	PsTPS-(−)lim	(−)-limonene synthase	ABA86248	Byun-McKay et al., 2006
	PsTPS-(−)αpin	(−)-α-pinene synthase	AAP72020	Byun-McKay et al., 2006
	PsTPS-1,8cin	1,8-cineole synthase	ADZ45499	Keeling et al., 2011
	PsTPS-(−)lin1	(−)-linalool synthase1	ADZ45501	Keeling et al., 2011
	PsTPS-(−)lin2	(−)-linalool synthase2	ADZ45502	Keeling et al., 2011
	PsTPS-(−)βphell1	(−)-β-phellandrene synthase1	ADZ45503	Keeling et al., 2011
	PsTPS-(−)βphell2	(−)-β-phellandrene synthase2	ADZ45504	Keeling et al., 2011

	PsTPS-(–)βphell3	(–)-β-phellandrene synthase3	ADZ45505	Keeling et al., 2011
	PsTPS-(–)βphell4	(–)-β-phellandrene synthase4	ADZ45506	Keeling et al., 2011
	PsTPS-(–)α/βpin	(–)-α/β-pinene synthase	ADZ45509	Keeling et al., 2011
	PsTPS-(+)3car	(+)-3-carene synthase	ADZ45511	Keeling et al., 2011
	PsTPS-(+)3car1R	(+)-3-carene synthase1(R)	HQ336798	Hall et al., 2010
	PsTPS-(+)3car1S	(+)-3-carene synthase1(S)	HQ336799	Hall et al., 2010
	PsTPS-(+)3car2R	(+)-3-carene synthase2(R)	HQ336800	Hall et al., 2010
	PsTPS-(+)3car3R	(+)-3-carene synthase3(R)	HQ336801	Hall et al., 2010
	PsTPS-(+)3car3S	(+)-3-carene synthase3(S)	HQ336802	Hall et al., 2010
	PsTPS-(+)sabR	(+)-sabinene synthase(R)	HQ336803	Hall et al., 2010
	PsTPS-(+)sabS	(+)-sabinene synthase(S)	HQ336804	Hall et al., 2010
Pseudotsuga menziesii (Pm)	PmTPS-terpinolene	terpinolene synthase	AAX07264	Huber et al., 2005
	PmTPS-(–)αpin/(–)camp	(–)-α-pinene/(–)-camphene synthase	AAX07267	Huber et al., 2005
**Outgroup:** *Physcomitrella patens*	PpTPS-entKS	*ent*-kaurene synthase	BAF61135	Hayashi et al., 2006
