# Supplementary material for: Transcriptome resources and functional characterization of monoterpene synthases for two host species of the mountain pine beetle, lodgepole pine (Pinus contorta) and jack pine (Pinus banksiana)
Source: BMC Plant Biol. 2013 May 16;13:80. doi: 10.1186/1471-2229-13-80 (PMC3668260; doi:10.1186/1471-2229-13-80)
Supplement: Additional file 7: Table S7 — Gene specific primers. [file 1471-2229-13-80-S7.pdf]

Table S7. Gene specific primers. Primers used for A) 5’ and 3’ RACE or to amplify full-length genes from B) Lodgepole pine and C) Jack pine cDNA or cDNA library. For In Fusion cloning into pET28b(+), the start codon ATG was removed, and the sequence (5’ AAATGGGTCGGGATCCG 3’) was added to the 5’ end of all forward primers and the sequence (5’CCGCAAGCTTGTCGAC 3’) was added at the 5’ end of all reverse primers.

A) Primers used in 5’ and 3’ RACE

| Species | Gene | 5’RACE primers (5’-3’) | 3’RACE primers (5’-3’) |
|---|---|---|---|
| Lodgepole pine (*Pinus contorta*) | *PcTPS-(−)βpin* | TGAGCTCCTCAACTGTTCCGAAGAG | Not required |
| | | CGCAGACATTTCATTTACGGTGTTGTAAACCAT | Not required |
| | *PcTPS-(−)βphell* | GGTGAGAGATTGTAACTCCCTCTTTTGTAACGAGTG | Not required |
| | | ATATGTCGGGTGAATGACAGTTGGGGGAAC | Not required |
| Jack pine (*Pinus banksiana*) | PbTPS-*(−)αpin* | TCTCAGCACGTTCCCGATAAGAAGGTTCC | AGGCGAGGAAAGGGACTGTATCAA |
| | PbTPS-*αterp* | GTCCATCGAGAGGATTGGTTGTAACA | Not required |
| | *PbTPS-monoTPS1/2* | TCCATTTGGCTTCTTGCATATGCGCAT | Not required |

B) Gene specific sequence of primers used to amplify candidate monoterpene synthases from lodgepole pine

| **Gene** | **Forward primer (5'-3')** | **Reverse primer (5'-3')** |
|---|---|---|
| *PcTPS-(+)αpin1* | ATGGCTCTGGTTTCTGCTGTCCC | TTACAAAGGCACAGTTTCAACCAC |
| *PcTPS-(–)αpin1* | ATGTCTCCTGTTTCTGTGATCTCGTT | TTATAAAGTGACAGGATCAATGACGGT |
| *PcTPS-(–)βpin1* | ATGGATTTAATATCTGTCTTACCGTCGAC | TTATAAAGGCACAGGTTCAAGGAG |
| *PcTPS-3car1* | ATGTCTCTTATTTCCGCTGTGCCGT | TTACATTAGCACGGGTTCGAGA |
| *PcTPS-(–)camp/(+)αpin1* | ATGGCTCTGGTTTCTGTTGCACCGTT | TTACAGAGGCACAGGTTCAAGG |
| *PcTPS-(–)βphell1* | ATGGCTCTGGCTCTGGTTTCTGTTGC | TTACAGAGGCACAGGTTCAAGG |
| *PcTPS-(–)βphell2* | ATGGCTCTGGTTTCTGTTGCACCGTT | TTACAGAGGCACAGGTTCAAGG |
| *PcTPS-αterp* | ATGGCTCTACTTTCTGTCGCGCCCTTG | TTACAGAGGCACAGGTTCAAGAACAG |

C) Gene specific sequence of primers used to amplify candidate monoterpene synthases from jack pine cDNA or cDNA library.

| Gene | Forward primer (5'-3') | Reverse primer (5'-3') |
|---|---|---|
| *PbTPS-(–)α/βpin1* | ATGGATTTAATATCT | TTATAAAGGCACAGG |
| *PbTPS-(+)αpin1* | ATGGCTCTGGTTTCT | TTACAAAGGCACAGT |
| *PbTPS-(–)αpin1* | ATGTCTCCTGTTTCTGTGATCTCGTTG | TTATAAAGTGACAGGATCAATGACGG |
| *PbTPS-(–)βpin1* | ATGGATTTAATATCTGTCTTACCGTCGGC | TTACAGAGGCACAGGTTCAAG |
| *PbTPS-(–)βpin2* | ATGGATTTAATATCTGTCTTACCGTCGG | TTATAAAGGCACAGGCTCAAG |
| *PbTPS-3car1* | ATGTCTCTTATTTCC | TTACATCAGCACAGG |
| *PbTPS-(+)3car2* | ATGTCTCTTATTTCCGCTGTGCCGT | TTACATTAGCACGGGTTCGAGA |
| *PbTPS-(–)βphell1* | ATGGCTCTGGCTCTGGTTTCTG | TTACAGAGGCACAGGTTCAAG |
| *PbTPS-αterp* | ATGGCTCTACTTTCTGTCGCGC | TTACAGAGGCACAGGTTCAAG |
| *PbTPS-mono1,2* | ATGGCTTTSGTTTCCGCTGTC | TTACAAAGGCACAGTTTCAACAAC |
